# EHMTI-0276. A novel mobile health application for patients with chronic headache

**DOI:** 10.1186/1129-2377-15-S1-D1

**Published:** 2014-09-18

**Authors:** K Abdel-Aziz, P Riding, S Woodham, J Blanco Rey, S Maddocks, L Wainwright, A Aljaaf, D Al-Jumeily, A Hussain, M Al-Jumaily, P Fergus

**Affiliations:** 1 Department of Neurology, Walton Centre For Neurology and Neurosurgery, Liverpool, UK; 2 Department of Computer Science, Liverpool John Moores University, Liverpool, UK

## Background

Headache is the commonest presenting complaint to neurology clinics across Europe and the developed world. However, within publically funded healthcare systems such as the UK’s National Health Service (NHS), long term follow-up in specialist clinics is not currently possible for all patients with chronic headache disorders. One potential solution to this challenge is to develop mobile health (mHealth) applications to allow specialists to monitor larger numbers of patients than would be possible within existing service models. We therefore aimed to develop a novel mobile application based system to allow remote monitoring of patients with chronic headache.

## System design

The system consists of separate interfaces for patients and clinicians. The patient interface, accessed through mobile devices, enables patients to keep headache diaries and assesses the impact of symptoms using quality of life scales. The web-based interface used by clinicians, charts patients responses in a user-friendly format. Longitudinal change in symptom severity is charted in relation to medication/lifestyle changes and other interventions. The treating clinician is also able to set alarm parameters for individual patients that would alert them that a patient needs review and if necessary expedite a clinic appointment.

## Conclusions

With patient compliance in mind, we have designed a user-friendly mobile application for remote monitoring of patients with headache. Such applications could potentially improve access to finite specialist headache services. Future work will aim to validate the use of this application in patients with headache.

No conflict of interest.

